# The role of centrifugal partition chromatography in the removal of *β*-asarone from *Acorus calamus* essential oil

**DOI:** 10.1038/s41598-022-26726-6

**Published:** 2022-12-23

**Authors:** Paweł Szczeblewski, Mateusz Wróblewski, Julia Borzyszkowska-Bukowska, Tetiana Bairamova, Justyna Górska, Tomasz Laskowski, Anna Samulewicz, Michał Kosno, Łukasz Sobiech, Justyna Teresa Polit, Wirginia Kukula-Koch

**Affiliations:** 1 Department of Pharmaceutical Technology and Biochemistry and BioTechMed Centre, Faculty of Chemistry, Gdansk University of Technology, Gabriela Narutowicza Str. 11/12, 80-233 Gdańsk, Poland; 2 Department of Cytophysiology, Faculty of Biology and Environmental Protection, University of Lodz, Pomorska Str. 141/143, 90-236 Lodz, Poland; 3 Catholic University High School in Tczew, Wodna Str. 6, 83-110 Tczew, Poland; 4 Agronomy Department, Faculty of Agronomy, Horticulture and Bioengineering, Poznan University of Life Sciences, Dojazd 11, 60-632 Poznan, Poland; 5 Department of Pharmacognosy with Medicinal Plants Garden, Medical University of Lublin, Chodzki Str. 1, 20-093 Lublin, Poland

**Keywords:** Bioanalytical chemistry, Mass spectrometry, Isolation, separation and purification, Analytical chemistry

## Abstract

*Β*-asarone is a phenylpropane derivative present in the rhizomes of *Acorus calamus*, that was proved to exhibit toxic effects in humans. Because of its presence the whole plant that is commonly used in traditional medicine for its sedative, anti-inflammatory, neuroprotective and other properties has limited application nowadays. In the study, qualitative and quantitative analysis of a collection of nine essential oil (EO) samples of European and Asian origin was performed. The final content of *β*-asarone in the tested samples ranged between 0.265 and 1.885 mg/mL. Having in mind a possible application of the EO as a biopesticide, this research aimed at the development of CPC-based purification protocol that could help remove *β*-asarone from EO. It was proved that the biphasic solvent system composed of n-hexane/EtOAc/MeOH/water, 9:1:9:1 (*v*/*v*/*v/v*) was capable of the removal of the toxic constituent in the CPC chromatograph operated in the ascending elution mode with 2200 rpm and a flow rate of 5 mL/min. The chromatographic analysis that lasted only 144 min effectively separated *β*-asarone (purity of 95.5%) and α-asarone (purity of 93.7%) directly from the crude *Acorus calamus* rhizome EO.

## Introduction

Maintaining high quality crop products parallelly with reduced synthetic pesticides content is the biggest challenge for the modern agriculture. The toxic pesticide residues accumulate in crop yields, soil and ground waters what triggers the development of pests’ and weeds’ resistance, but simultaneously, affects soil microbiome and well-being of people and animals. However, the plant derived products of the secondary metabolism which include, *inter alia*, volatile components/ essential oils (EO) usually recovered from plant matrix by hydrodistillation seem to be a promising alternative for the synthetic pesticides used so far. Additionally, the EOs were proved to be efficient both in the prevention and treatment of different diseases^1^. Formerly their antibacterial, antifungal and insecticidal properties were discussed in the scientific literature^2^. Furthermore, their strong allelopathic properties such as the influence on germination and seedling growth should not be underestimated. The richness and variety of EOs components affects their diversified mechanisms of action and consequently hampers the resistance development in pathogens, pests, and weeds towards the oils. In the end, the effortless biodegradability of EO does not pose a risk to the environment.

**Figure 1:**
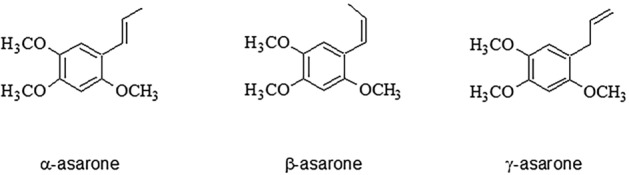
The isomers of asarone found in *Acorus calamus* EO.

The EO that exhibits dualistic, and thus intriguing properties is the calamus oil. It is extracted from rhizomes of sweet flag (*Acorus calamus* L.) belonging to Araceae botanical family. The leading constituents of calamus EO are phenylpropane derivatives (biosynthesized via the shikimate pathway), including propenylic isomers: a mixture of *trans* form of *α*-asarone, *cis* form of *β*-asarone ((E)-/(Z)-1,2,4-trimethoxy-5-prop-1-enylbenzene) and an allylic derivative *γ*-asarone (1,2,4-trimethoxy-5-prop-2-enylbenzene) (see Fig. 1). Methyleugenol, (Z)-methylisoeugenol, and elemicin are the remaining phenylpropenes detected in the calamus oil. The second group of EO constituents are terpenes: monoterpenes (*α*- and *β*-pinene, camphene, myrcene, limonene) and sesquiterpenes (cadinene, acorone, acorenone, calamine). Also, the presence of tannins, choline, and fatty acids in its rhizomes was previously described^3–6^.

The amount of phenylpropenes varies depending on the ploidy of *A. calamus* variant. In North America and in parts of Asia (Siberia) a diploid form (2n = 2x = 24) of the sweet flag may be found, which was proved to contain only 2% of EOs with no or trace amounts of *β*-asarone in its composition. *A. calamus* plants growing in Europe and temperate regions of Asia are primarily triploids (2n = 3x = 36) containing more EOs (3%) and little amounts of *β*-asarone (5–19%) in the EO. The third, tetraploid variety (2n = 4x = 48) that is most commonly used for the industrial production of calamus oil is widespread in East Asia, Japan and India and contains up to 6% of EOs and the largest amount of *β*-asarone (70–96%) in the EO from the rhizomes^7,8^.

For many years sweet flag extracts remained substances of interest due to their their medicinal properties. Calamus preparations exhibit neuroprotective, antidepressant, anticonvulsive, sedative, hypnotic, anxiolytic, memory enhancing, antiasthmatic, antihyperlipidemic, anticholestatic, antithrombotic, antiinflammatory, antioxidant, anticarcinogenic, antimicrobial and insecticidal properties^9–12^. Even if the traditional usage of the plant in medicine, fragrance industry and foods has been wide, the exact *modus operandi* of calamus phytochemicals is still unexplored. Also, the contradictory reports on the toxicological safety of calamus oil for humans still need a final conclusion. Some authors mention cardiotoxicity, hepatotoxicity, reproductive toxicity, mutagenicity and carcinogenicity of *β*-asarone^7^. Based on the reported adverse effects, legal regulations for the use of *β*-asarone have been established. Within the European Union, the use of *β*-asarone as a pure substance or in the form of the tetraploid calamus essential oil for flavoring purposes is currently prohibited. In the United States of America, the use of calamus oils and its preparations is utterly disallowed. Several scientific reports on the therapeutic application of *β*-asarone, like memory enhancing properties^13^ or antimicrobial action^14^ re questionable in the light of the regulations described above. The observed tendency in the number of scientific manuscripts in the Scopus database shows a decrease in the number of articles concerning *Acorus calamus*, while the number of scientific papers on *β*-asarone itself stays on the same level.

Despite the toxicological data presented above, calamus EO still bears potential to be used as a biopesticide^15^. It was proved that calamus oil contains a number of important active substances (other than *β*-asarone) with a synergistic effect when in mixture^16–19^. In the light of these findings, a good solution, safe for farmers and customers, might be to develop a method of effective removal of *β*-asarone from calamus oil aspiring to the role of a biopesticide—to be able to use it with no risk for poisoning of humans.

Classical isolation methods used to separate EOs components, like HPLC or TLC chromatography, even if they are offering several advantages like the reproducibility of results, automation, quantitative determination, short analysis time, they are not suitable for the analysis of all secondary metabolites or in the purification on semi- or preparative scale^20^. One should mention here an irreversible adsorption on the chromatographic bed, which results in the sample loss and the damage of expensive preparative columns, tailing of compounds of interest on the stationary phase which decreases the purity of the isolates, or small loading capacities which limit the application of these techniques to the industrial scale isolation protocols. Also, the high consumption of solvents makes the classical separation process unprofitable and failing the requirements of green chemistry practice.

In the scientific literature there are only a few examples of *β*-asarone isolation. Previously, McGaw and co-investigators introduced a two-step fractionation (column chromatography and preparative thin layer chromatography) of the crude ethanol extract from *Acorus calamus* roots, rhizomes and leaves. In the proposed protocol they obtained 15.6 mg of the component from 1.14 g of extract^14^. In another work the same compound was purified from the rhizome volatile oil with chloroform: petroleum ether (4:1 *v/v*) using a silica gel chromatographic column^13^. The two mentioned above techniques were based on the application of classical chromatographic tools in the recovery of *β*-asarone and are bearing several limitations. The recovery rate and the loading capacity of these protocols was low, whereas the procedure tedious and the selectivity poor. Both techniques needed high volumes of solvents to be run, were time consuming, and the cited sources did not provide any information about the purity of the isolate. Based on these observations the authors of this manuscript found it important to search for a new isolation protocol that would provide high purity *β*-asarone directly from the crude EO sample to be either removed or isolated from EO. Easy scale-up, low solvents consumption and short analysis time would be preferred for future application in biopesticide production as well as in other calamus preparations.

Centrifugal Partition Chromatography (CPC) is a technique that is devoid of these drawbacks. This modern separation tool is the hydrostatic type of countercurrent chromatography, which uses the phenomenon of liquid–liquid partitioning of compounds between two non-miscible phases that stay at equilibrium^21^. One phase (stationary one) is retained in the column, while the other one (mobile phase) is pumped through it. Both immiscible liquids are in constant contact, emulsifying and separating repeatedly, creating the phenomenon of theoretical plates. The retention mechanism of stationary phase is determined by hydrostatic force, formed by the centrifugal field in the rotor in one-axis centrifuge^22,23^. The separation of constituents of a mixture is obtained as a function of partition coefficient Kd, defined as the concentration of target compound in the stationary phase divided by the concentration in the mobile phase^24^.

CPC is able to provide repetitive and selective separation conditions for the purification of single metabolites from plant extracts—also on preparative scale. High selectivity of applied methods, low solvent consumption, high sample loading capacity and lack of solid support that prevents sample loss is conducive to recover sufficient quantities of natural products even from complex plant extracts^25,26^. However, the scientific literature on the analysis of essential oils by counter current chromatography is limited. Interestingly, according to our knowledge only four scientific works are focusing on the separation of essential oils using Centrifugal Partition Chromatography were traceable^27–30^, while the remaining majority concerned the HSCCC technique. At this point it is worth mentioning that the CPC technique creates more advantageous conditions for a large-scale separations than the HSCCC technique.

Therefore the aim of this study is to develop precise single-step methodological conditions that would provide calamus oil free from *β*-asarone to be used on industrial scale. For this purpose CPC chromatography will be used to give hope for the successful exclusion of this potentially toxic phenylpropane derivative and possible application of the *β*-asarone-free EO as biopesticide in the future. Also, the determination of *β*-asarone content in the personally collected and commercially available EO samples will be performed with help of the HPLC-DAD and HPLC-ESI-QTOF-MS/MS techniques to confirm the actual content of the terpene of interest in the European and Asian samples and to emphasize the need for its removal.

## Results and discussion

### Essential oil composition by HPLC-ESI-QTOF-MS/MS

**Figure 2:**
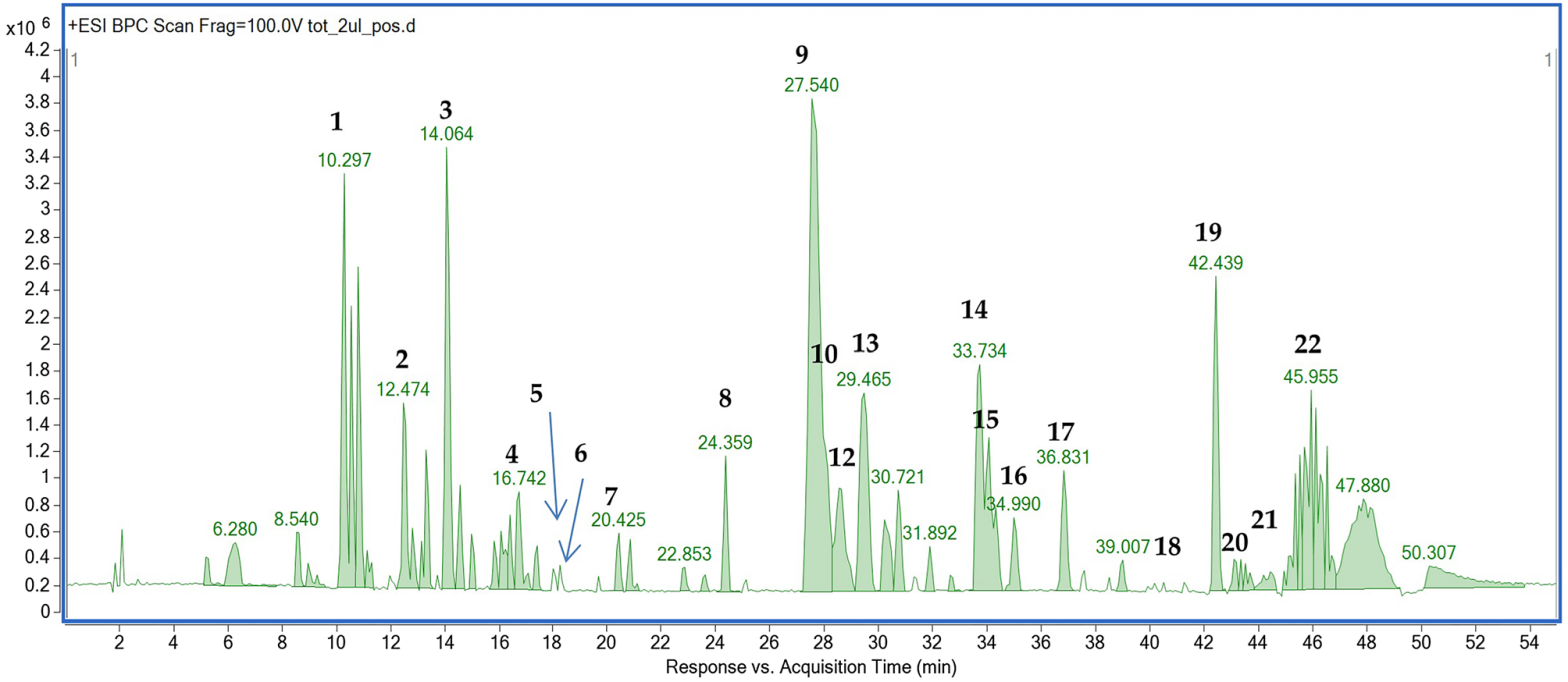
The total ion chromatogram (HPLC–MS) in positive ionization mode of the commercial essential oil sample obtained from the rhizomes of *Acorus calamus*.

The performed HPLC-ES-QTOF-MS/MS analysis of the EO samples led to the identification of 22 compounds out of ca. 40 signals present in the mass chromatogram. The applied instrument was sensitive enough to show clear mass spectra and sharp peaks of unpolar constituents of *Acorus calamus* rhizomes using a standard RP-18 chromatographic column. The composition of a sample EO and the list of tentatively identified components that was prepared in consideration with literature results on different *Acorus spp.* are presented in the Fig. 2, in Table 1 and also Table S1 (Supplementary File).

**Table 1:** The putative identification of secondary metabolites present in the essential oil obtained from the tested rhizome of *Acorus calamus*.

No	Ion (+/−)	Rt (min)	Molecular formula	*m*/*z* calculated	*m*/*z* experimental	Delta (mmu)	DBE	MS/MS fragments	Proposed compound	References
**1**	[M + H]^+^	10.3	C_12_H_16_O_4_	225.1121	225.1136	5.42	5	210, 193, 165	**Isoacoramone isomer1**	Sinha et al.^31^
**2**	[M + H]^+^	12.4	C_12_H_16_O_4_	225.1121	225.1131	− 4.3	5	210, 193, 165	**Isoacoramone isomer2**	Sinha et al.^31^
**3**	[M + H]^+^	14.1	C_10_H_12_O_4_	197.0808	197.0811	− 1.35	5	169,154,138	**Asaronaldehyde**	Jaiswal et al.^32^
**4**	[M + H]^+^	16.7	C_6_H_12_O_7_	197.0656	197.0662	− 3.17	1	169, 154, 138	**Gluconic acid**	Strzepek-Gomolka et al.^33^
**5**	[M + H]^+^	18.0	C_11_H_14_O_3_	195.1016	195.1014	0.88	5	–	**Propioveratrone**	Jaiswal et al.^32^
**6**	[M + H]^+^	18.2	C_17_H_12_NO_3_	279.0890	279.0812	− 2.73	12.5	–	**Tatarine A**	Zhang et al.^34^
**7**	[M + H]^+^	20.1	C_10_H_10_O_2_	163.0754	163.0760	− 3.97	6	–	**Safrol**	Jaiswal et al.^32^
**8**	[M + H]^+^	24.6	C_15_H_22_O_2_	235.1693	235.1702	− 4.03	5	217, 179, 161	**Acoronene**	Yang et al.^35^
**9**	[M + H]^+^	27.5	C_7_H_6_O_3_	209.1172	209.1163	4.42	5	168,153	**2-Allyl-5-ethoxy-4-methoxyphenol isomer 1 (β-asarone)**	Jaiswal et al.^32^ Yang et al.^36^
**10**	[M + H]^+^	28.3	C_15_H_22_	203.1794	203.1799	− 2.34	5	161, 147, 133	**Calamenene**	Jaiswal et al.^32^
**11**	[M + H]^+^	28.6	C_11_H_14_O_2_	179.1067	179.1071	− 2.49	5	164, 151	**Methyl-eugenol**	Jaiswal et al.^32^
**12**	[M + H]^+^	28.6	C_7_H_6_O_3_	209.1172	209.1179	− 3.26	5	168, 153	**2-Allyl-5-ethoxy-4-methoxyphenol isomer 2 (γ-asarone)**	Jaiswal et al.^32^ Yang et al.^36^
**13**	[M + H]^+^	29.4	C_7_H_6_O_3_	209.1172	209.1183	− 5.19	5	194, 181	**2-Allyl-5-ethoxy-4-methoxyphenol isomer 3 (α-sarone)**	Jaiswal et al.^32^ Yang et al.^36^
**14**	[M + H]^+^	33.6	C_12_H_16_O_4_	225.1121	225.1136	− 6.54	5	210, 193, 165	**Aspidinol**	Jaiswal et al.^32^
**15**	[M + H]^+^	33.9	C_15_H_22_O	219.1743	219.1753	− 4.39	5	177, 159, 121	**Squamulosone isomer 1**	Jaiswal et al.^32^
**16**	[M + H]^+^	34.7	C_22_H_28_O_6_	389.1959	389.1972	− 3.44	9	–	**Surinamensin**	Jaiswal et al.^32^
**17**	[M + H]^+^	36.8	C_15_H_22_	203.1794	203.1811	− 8.27	5	161, 147, 133	**Calamenene isomer**	Jaiswal et al.^32^
**19**	[M + H]^+^	41.4	C_15_H_24_O_2_	237.1849	234.1848	0.45	4	219, 135	**Acorusnol**	Yannai^37^
**18**	[M + H]^+^	42.8	C_15_H_22_O	219.1743	219.1752	− 3.93	5	177, 137, 121	**Squamulosone isomer 2**	Jaiswal et al.^32^
**20**	[M + H]^+^	42.4	C_22_H_24_	205.1951	205.1953	− 1.09	4	165, 149, 125	**Calarene**	Loying et al.^38^
**21**	[M + H]^+^	44.1	C_22_H_24_	205.1951	205.1961	− 5.01	4	149, 121, 81	**α-Cedrene**	Jaiswal et al.^32^
**22**	[M + H]^+^	45.9	C_15_H_24_O	221.1900	221.1920	− 9.12	4	153, 83	**(+)-Shyobunone**	Jaiswal et al.^32^

DBE, double bond equivalent; delta, error of measurement; Rt, retention time; Ion, ionization mode.

Among the representatives of different classes of compounds, asarones belonged to the most abundant components of the tested essential oils, with *β*-asarone as the leading compound. The identified constituents—terpenes and other asarone-related phenylpropanoids, were previously mentioned by other authors in *Acorus calamus* and *Acorus tatarinowii* species, as shown in Table 1.

### Quantitative analysis of asarone content in EO by HPLC-DAD

**Figure 3:**
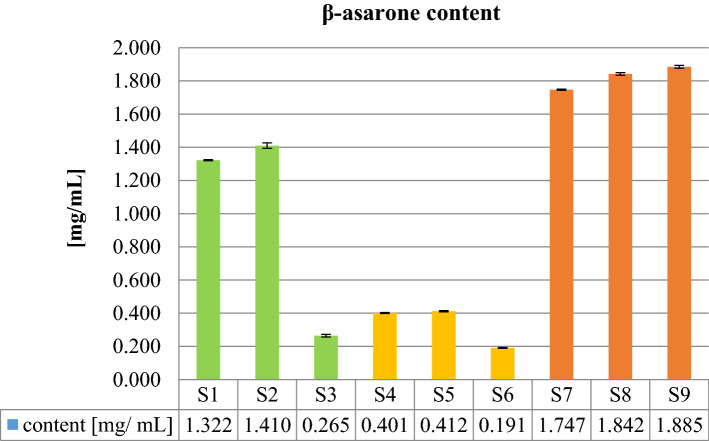
The *β*-asarone concentration in the analysed EOs.

The application of a liquid chromatography in the analysis of phenylpropanoids’ content in the EO of *A. calamus* brought successful results. The HPLC-DAD based quantitative analysis of the asarones visualized the three forms of asarones: *α-*, *β*- and *γ*-asarone. These compounds were eluted from the column at 14.0, 11.5 and 12.8 min, respectively (see Fig. 7A). The quantitative analysis of asarones in the tested samples revealed marked differences between the samples. Considering the relative percentage content, *β*-asarone was found to be the major phenylpropanoid visible in the HPLC-DAD chromatogram, followed by α-asarone and γ-asarone in all tested samples (see Table 2). Interestingly, the total concentration of the *β*-asarone differed between the samples (see Fig. 3).

**Table 2:** The relative amount [%] of asarones in the analysed EOs.

Source	Rhizomes of natural accessions			Commercially available rhizomes			Commercially available EOs		
Code	S1	S2	S3	S4	S5	S6	S7	S8	S9
**Asarone content [%]**									
α	9.9	8.4	8.7	10.2	9.0	14.2	4.7	6.2	4.8
β	89.2	90.9	90.8	88.7	90.0	84.7	94.1	92.6	94.0
γ	0.8	0.8	0.5	1.1	1.0	1.2	1.2	1.2	1.1

Its content was calculated as 1.32–1.41 mg/mL of the EO in the samples S1 and S2, 0.27 mg/mL in the sample S3, 0.19–0.41 mg/mL in the EOs from the commercially available rhizomes (samples S4-S6) and 1.75–1.89 mg/mL in the commercially available EOs from *A. calamus* (Samples S7-S9). Interestingly, the content of *β*-asarone differed depending on the place of collection of the rhizomes. The quantity of this compound in the Sample S3 that was collected in the central Poland in Małczew was significantly lower from the samples S1 and S2 (see Fig. 3). It could be explained by the presence of different ploidy plants on the area of Poland.

The obtained quantitative results for *β*-asarone in calamus oil are in line with some previously published data. A thorough study on its content in Finnish and Czech samples authored by Dušek et al.^39^ proved that this phenylpropanoid was present in the oil at the quantity of 9.7–21.3% that resembles the concentration of 0.97–2.13 mg/mL. In general, the samples from Finland contained lower quantities of *β*-asarone in comparison to the Czech samples, but still its content was higher than in the herein investigated samples from the central Poland.

Also, in the herein presented study the content of *β*-asarone was high in the commercially available EOs. The concentration of this phenylpropanoid in the samples S7-S9 resembled its content in the samples S1-S2.

The quantitative analysis of *β*-asarone content in the analysed samples brought to light an interesting issue, namely the geographical occurrence of different ploidy *A. calamus* plants. Even if Poland was expected to host triploids containing more EOs with small share of *β*-asarone, as mentioned in the introduction section, the quantitative analysis showed marked quantity of this component in the calamus oil similarly to the, widely distributed in Asia, tetraploid variety. The calculated *β*-asarone content is close to S9 sample of the Indian origin. Based on the obtained results, we could assume there are different ploidy plants in European grounds.

According to Raal and co-investigators^40^ *β*-asarone was also the major constituent of the *A. calamus* rhizome essential oil that was obtained from the specimen grown in Estonia. The compound of interest constituted 85.3% in one sample—that was identified as a tetraploid sweet flag and 9.3–10.2% in other samples that were possibly triploids. These data confirmed the possibility to find in Europe different ploidy specimen growing in nature. The authors of this publication underlined that their data from 2016 mentioned for the first time the presence of *β*-asarone-rich chemotype of sweet flag in Europe. For sure, these specimens can also grow on the territory of Poland, as proved in our study.

### Chemometric assessment

Since various extracts from *Acorus calamus* were collected at different dates and using different sources and techniques, for chemometric analysis it was decided to examine the relative concentrations of *α-*, *β*-, and *γ*-asarone in each sample, instead of the absolute values (Table 2).

**Figure 4:**
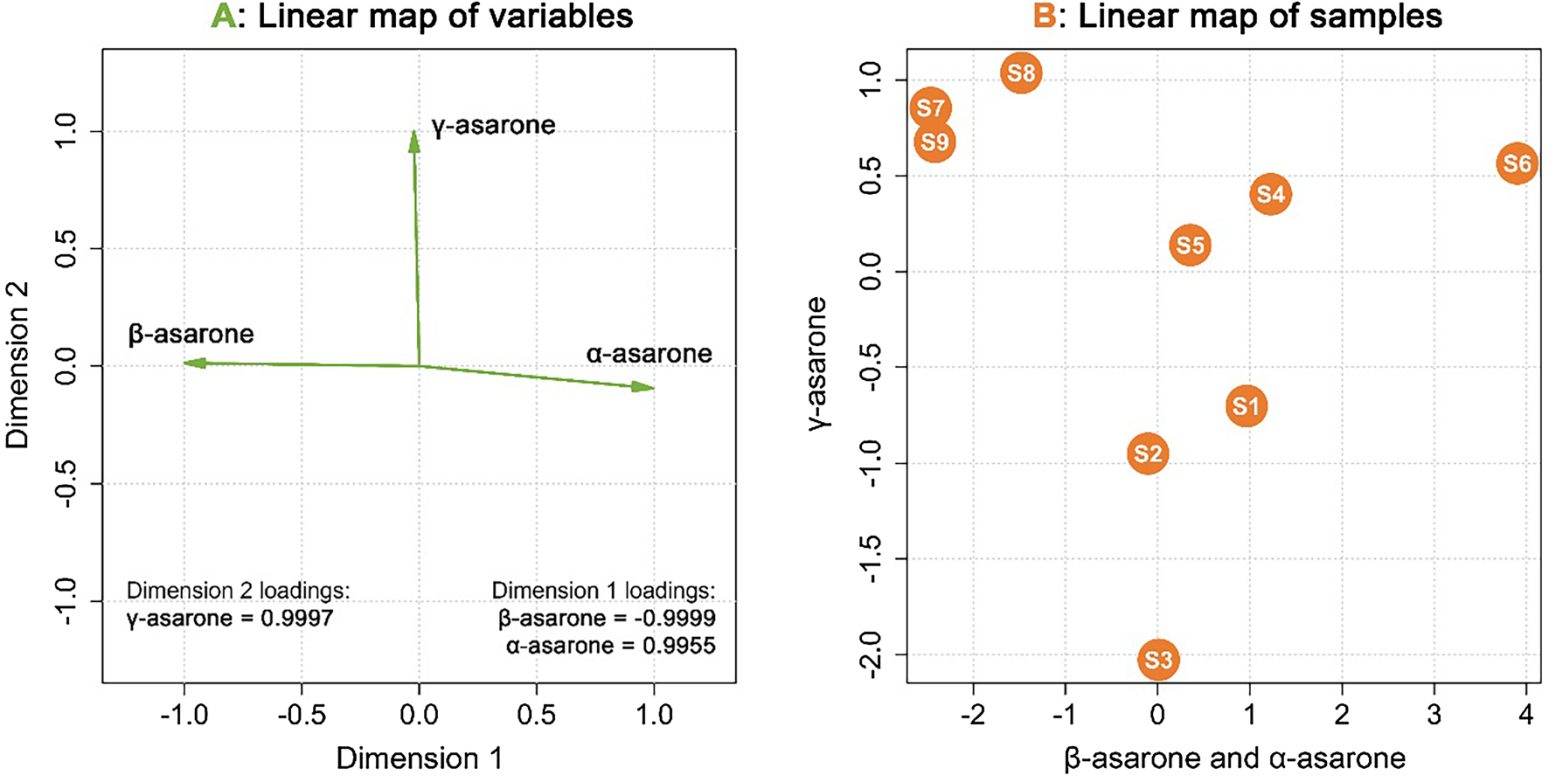
The linear map of original variables in the space of the two relevant dimensions of the discussed problem (**A**) and the linear map of samples in the same coordinate system (**B**).

Principal Component Analysis (PCA) has revealed that the studied dataset was only two-dimensional, since the first two principal components extracted almost 100% (~ 99.99998%) information of the original data matrix. It was revealed that the relative concentration of α-asarone was almost perfectly, yet reversely correlated to the relative concentration of *β*-asarone, which again confirmed that both compounds have common origin and possibly could be transformed from one to another via enzymatic or light-induced E/Z isomerization. Hence, the information on α- and *β*-asarone constituted the first dimension of the examined dataset after applied VARIMAX rotation algorithm. The second dimension of the data contained solely information on the relative concentration of γ-asarone, which displayed no correlations to its α- and *β*- counterparts (Fig. 4).

Figure 4 presents a linear map of samples in the space of the two dimensions established above. Fresh samples collected from northern Poland (S1 & S2) exhibited lower than average relative concentrations of γ-asarone with and medium relative concentrations of α- and *β*-asarone. The S3 sample, collected from central Poland presented much lower relative amounts of γ-asarone in comparison to the rest of the sources. Samples S4 and S5, coming from commercially available rhizomes, displayed average relative concentrations of all three traced asarones. In contrary, sample S6, while maintaining relative amounts of γ-asarone similar to the ones of S5 and S6, exhibited highest—by far—relative concentration of α-asarone and, therefore, lowest relative concentration of *β*-asarone. Finally, the commercially available essential oils (S7-S9) presented the highest relative amounts of *β*- and γ-asarones and the lowest relative amounts of α-asarone in the whole studied group.

**Figure 5:**
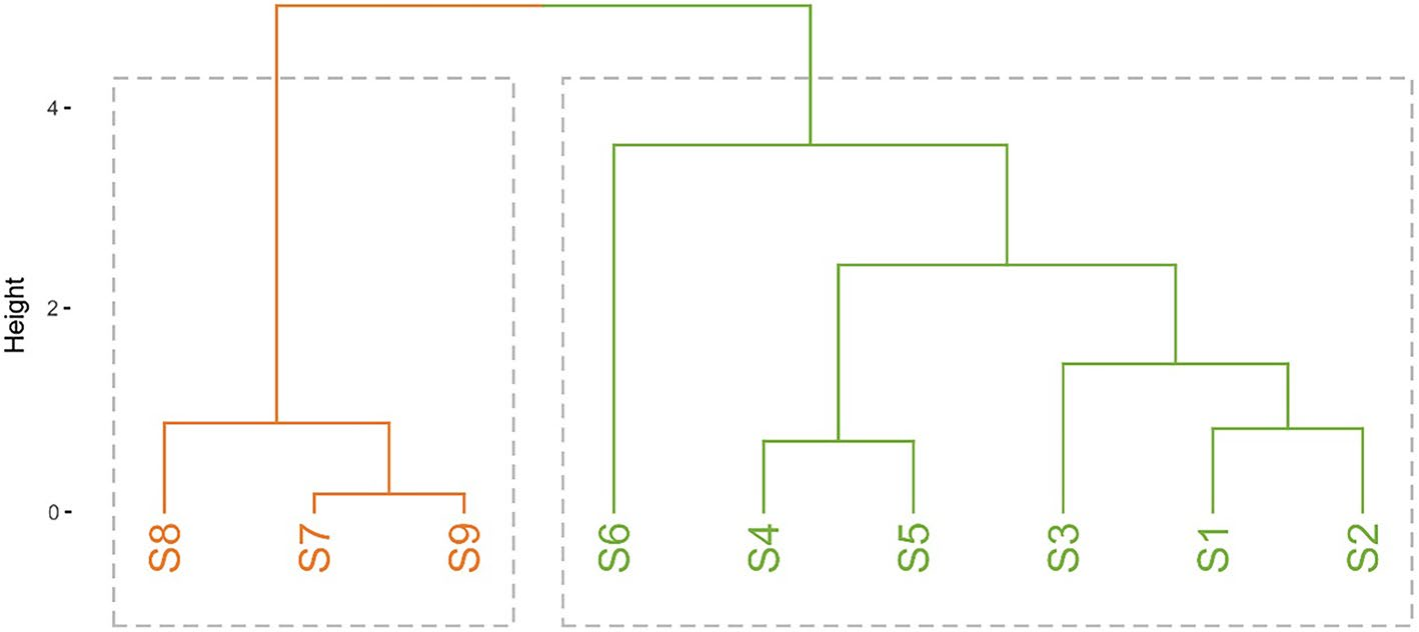
Cluster dendrogram, drawn on the basis of the Euclidean distances between studied samples, concerning relative concentrations of *α*-, *β*- and *γ*-asarone.

Cluster dendrogram (Fig. 5), prepared on the basis of the relative concentrations of all three studied asarones, has furtherly proved that extracts obtained from collected and commercially available rhizomes might be considered similar, with sample S6 being a potential outlier in the group—as PCA has proven, due to significantly different *α/β* ratio in comparison to samples S1-S5. Nevertheless, in comparison to extracts S1-S6, samples S7-S9, coming from commercially essential oils, exhibited fundamentally different asarone compositions, hence formed a separate cluster on the diagram.

### CPC method of removing *β*-asarone from *A. calamus* EO

Centrifugal partition chromatography (CPC) as an emerging purification technique is used in the separation process of natural products, protein mixtures, synthetic drugs, APIs and others. It belongs to green techniques due to a limited usage of solvents in the separation process, but most interestingly, it is more commonly selected by the industrial players as a technique reaching industrial-scale solutions^41^.

CPC chromatographs are used for industrial concentration and purification of several groups of natural products or single compounds, e.g. omega-3 fatty acids^42^ or acidic cannabinoids from *Cannabis sativa*^43^. High selectivity and repeatability of the CPC-based analyses is certainly a strong advantage of this technique, however, its good operation must be based on a proper selection of the biphasic solvent system that should harmonize with the analysed sample and be selective towards the single constituents of the mixture.

#### Solvent system selection

The selection of the appropriate biphasic system plays a key role in counter-current chromatography, and therefore, this step should commence any method development of the separation process. Herein, an appropriate solvent system was chosen according to the partition and separation factors (Kd and α). Due to the hydrophobicity of target compounds, a series of low-polar solvent systems composed of n-hexane, ethyl acetate, methanol and water in different volume ratios (HEMWat/ Arizona system) were tested to provide an efficient separation of the target compounds. The Arizona system was also selected as there is no need for preparing various biphasic solvent systems in separating funnels (Gilson Glider software combined with a 4-channel pump is able to mix relative portions of the selected solvents directly in the apparatus). This aspect is important in case of pilot and industrial-scale application of developed method as it lowers the consumption of solvents necessary for the separation process. Table 3 summarizes distribution coefficients (Kd) and separation factors (α) calculated for target compounds in the different volume ratios of HEMWat system.

**Table 3:** Partition coefficient values calculated for some biphasic solvent systems and related selectivity for α-asarone and *β*-asarone.

System	n-Hex/EtOAc/MeOH/H2O solvent system (v/v/v/v)	Kd		α
		α-asarone	β-asarone	
1	1:1:1:1	0.15	0.28	1.87
2	6:1:6:1	1.34	1.26	1.06
**3**	**9:1:9:1**	**1.94**	**1.70**	**1.14**
4	19:1:19:1	2.48	2.12	1.17

Selected solvent system is in bold.

Two of the tested two-phase solvent systems (system 2 and 3) met the first criterion of efficient separation with Kd in the range of 0.5–2^44^. Among the two remaining systems, system 3 and 4 were selected for further evaluation on CPC chromatograph based on their beneficial differences between Kd values of the analysed components.

#### CPC separation

First, the experiment was run at different rotation speed of the column that was within a range of 1000–2300 rpm, at a constant flow rate of 5 mL/min to evaluate the impact of rotation speed on the selectivity. Finally 2200 rpm setting was selected as the most advantageous one, giving the best selectivity towards asarones with no drastic increase in the operating pressure. The selected relatively high rotation speed kept together with the flow rate of 5 mL/min generated the pressure of 50 bar that is half of the maximum operational pressure in CPC chromatographs. Sustaining a quicker flow rate as 5 mL/min markedly reduced the analysis time. During the optimisation time system No 4 was rejected, as it did not bring better selectivity towards both asarones, but only prolonged the analysis time of 20 min.

**Figure 6:**
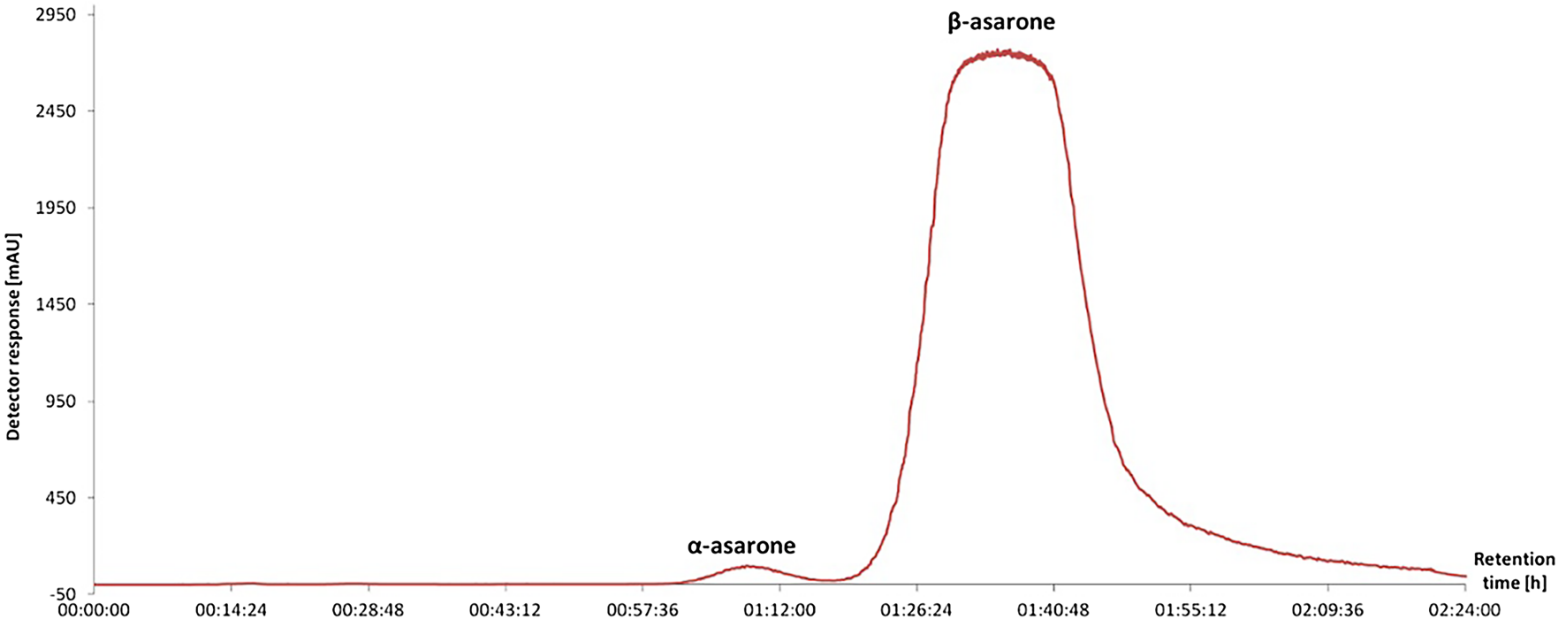
The CPC chromatogram (CPC-DAD) of the essential oil obtained from the rhizomes of *Acorus calamus* by hydrodistillation in the Clevenger apparatus. Monitored wavelength, λ = 254 nm.

As a result of the CPC-based fractionation of *Acorus calamus* EO using the solvent system composed of n-Hex/EtOAc/MeOH/H_2_O (9:1:9:1 *v/v/v/v*), the complete separation of both *α-* and *β*- asarones from the essential oil was obtained in a short time of 144 min in the elution mode (see Fig. 6 for the chromatogram from CPC chromatograph). The calculated Sf value of 72% is consistent with the stationary phase retention suggested for counter-current separations^45^. It should be mentioned, that for all CPC columns, the Sf maximum value is necessarily much lower than 100% with the maximum value reaching 80%. Such values are dictated by the construction of the hydrostatic apparatus^46^.

**Figure 7:**
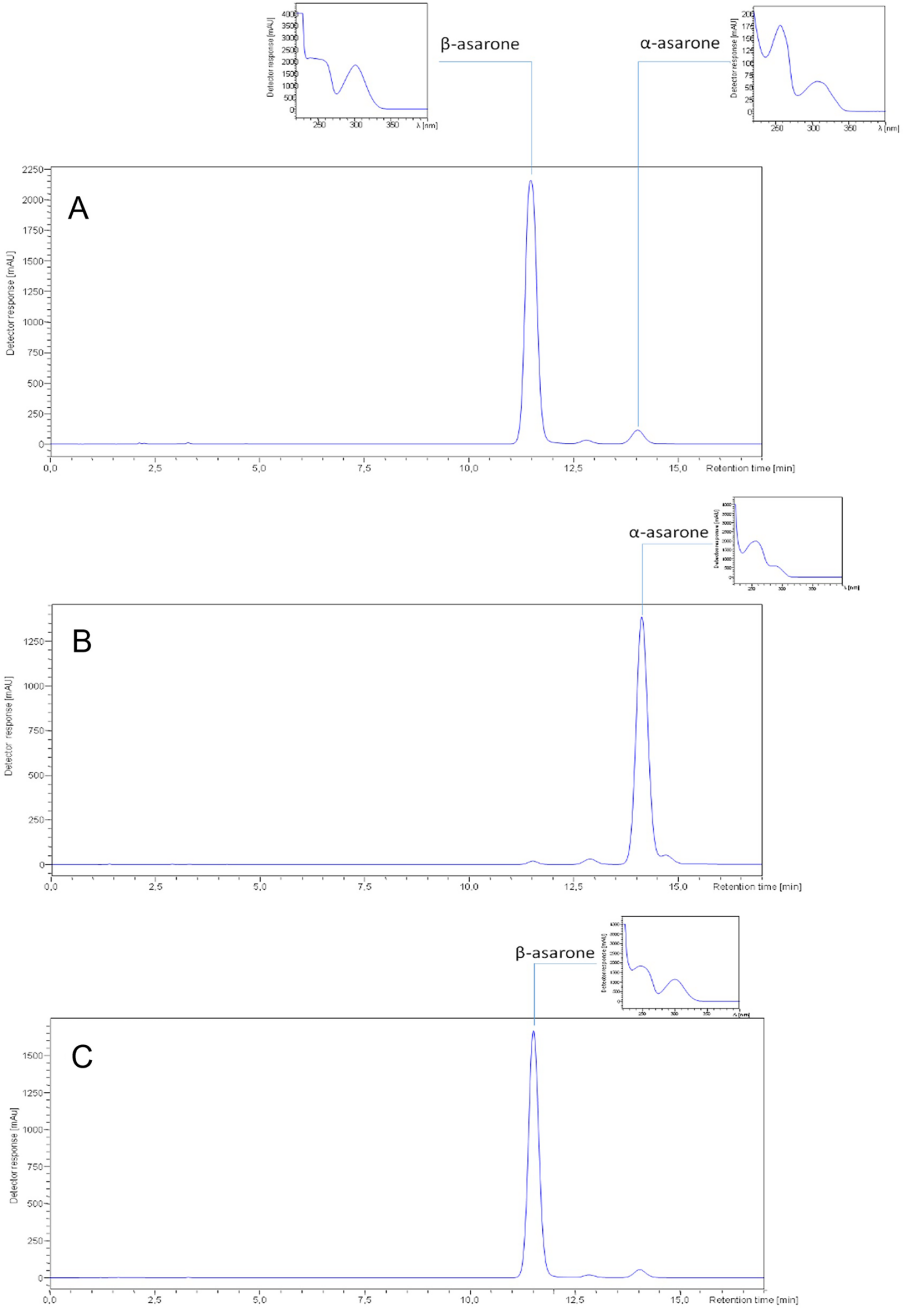
(**A**) HPLC-DAD chromatogram of the standard solution of *Acorus calamus* EO and UV spectra of *α*- and *β*-asarone; (**B**) HPLC-DAD chromatogram and UV spectrum of the α-asarone fraction obtained by CPC separation; (**C**) HPLC-DAD chromatogram and UV spectrum of the *β*-asarone fraction obtained by CPC separation.

The fractions containing asarones were separated, whereas all remaining fractions were collected together. After the completion of the fractionation process the purity check of the isolates was performed using the HPLC-DAD instrumentation, compared with the total EO sample (see Fig. 7) and the purity of asarones was calculated as 93.7% for α- and 95.5% for *β*- asarone (see Table S4 in the Supplementary File). Interestingly, both compounds were eluted from the column in the elution mode, so as the remaining constituents of the essential oil could be easily pushed out from the column with a higher flow rate of 30 mL/min. This way, the performed fractionation of the EO succeeded in the separation of asarones from the remaining constituents of the oil. Collecting together all fractions except those containing asarones provides EO with no presence of asarones bearing toxic properties.

The elaborated purification protocol is the first one dedicated to the hydrostatic counter-current instrumentation, like CPC chromatographs. Previously, successful trials to recover asarones were performed on a different species, namely on *Acorus tatarinowii* EO and were published by Wang and co-investigators. The researchers used a different type of chromatograph in their study—an HSCCC chromatograph that is operated in the hydrodynamic mode. The used mixture of n-hexane–ethyl acetate–methanol–water (1:0.2:1:0.3, *v/v*) fractionated 1.0 g of the essential oil. The analysis was conducted for 6 h to obtain *α*- and *β*-asarones in the eluate. The authors used 800 rpm rotation speed of the column and the flow rate of only 1.5 mL/min. The selected parameters had impact on an increased purification time and small sample loading. Even if the previously described purification protocol provided high purity asarones, the application of HSCCC chromatographs is limited to analytical and semi-preparative conditions due to high pressure that is generated during the run.

Asarone-free calamus oil still shows potential as an eco-friendly green biopesticide or as an bioherbicide against weeds as it contains a mixture of many biologically active chemicals. Identified substances such as calarene, shyobunone, safrol, cedrene were detected also in other EOs exhibiting bactericidal, fungicidal, insecticidal, repellent or allelopathic activities, inhibiting germination different species^47–51^. According to Liu and co-investigators^52^ α-asarone, methyleugenol and (*E*)-methylisoeugenol isolated from calamus oil were the strongest fumigants/insecticides and were found toxic against booklouse *Liposcelis bostrychophila*. In another study Chen et al.^47^ underlined a strong impact of shyobunone and isoshyobunone isolated from calamus oil on the development of *Lasioderma serricorne* (LS) and *Tribolium castaneum* (TC)*.* The isolates were characterized by a strong contact toxicity against LS and TC. The calculated LD50 values against LS adults were 20.24 and 24.19 µg/adult specimen, respectively. The oil and the isolates were found to be strongly repellent already after 2 h of treatment.

The above examples show marked importance of calamus oil other than *β*-asarone. Based on this observation, the removal of this toxic component from the EO is logic and important especially these days, when new sources of repellent compounds that are neutral to environment are needed. Prior to extensive production, properties of the oil deprived of *β*-asarone, parallelly with the one containing *β*-asarone, must be thoroughly evaluated. Their impact on human cell lines, organisms present in the agrocenosis as well as germination and seedling development of various species should be analysed.

## Conclusions

Chromatographic techniques are important tools suitable for the evaluation of plant extracts’ composition. They are capable of fingerprinting^53^ the extracts and also isolating single components from the matrix^54^. Also in this study the qualitative analysis of the tested EO samples performed by HPLC and HPLC–MS led to the identification of 22 metabolites including *β*-asarone as a major constituent. According to our study, the content of *β*-asarone in the European origin EO depends on the location and can resemble the high content of the compound observed in tetraploid specimen from Asia. Having the widespread use of calamus oil in traditional medicine on the one hand, and the data on the toxicity of *β*-asarone on the other, it is very important to implement a procedure of *β*-asarone elimination from plant material. The proposed herein technique of removing *β*-asarone from the rhizome oil of *Acorus calamus*—the CPC chromatography—gives hope for the potential use of calamus oil as a potential bioherbicide—that could be safe for the environment and humans. Previously published data indicate strong repellent activity of other ingredients than β-asarone present in calamus oil, so its removal will not affect the total biocidal effectiveness. The CPC method proposed in this paper is simple, quick, meets the requirements of green chemistry practice, due to the relative low solvent consumption and promises simple upscaling conditions that would provide high quantities of *β*-asarone free calamus oil.

## Methods

### Plant material and extraction procedure

**Figure 8:**
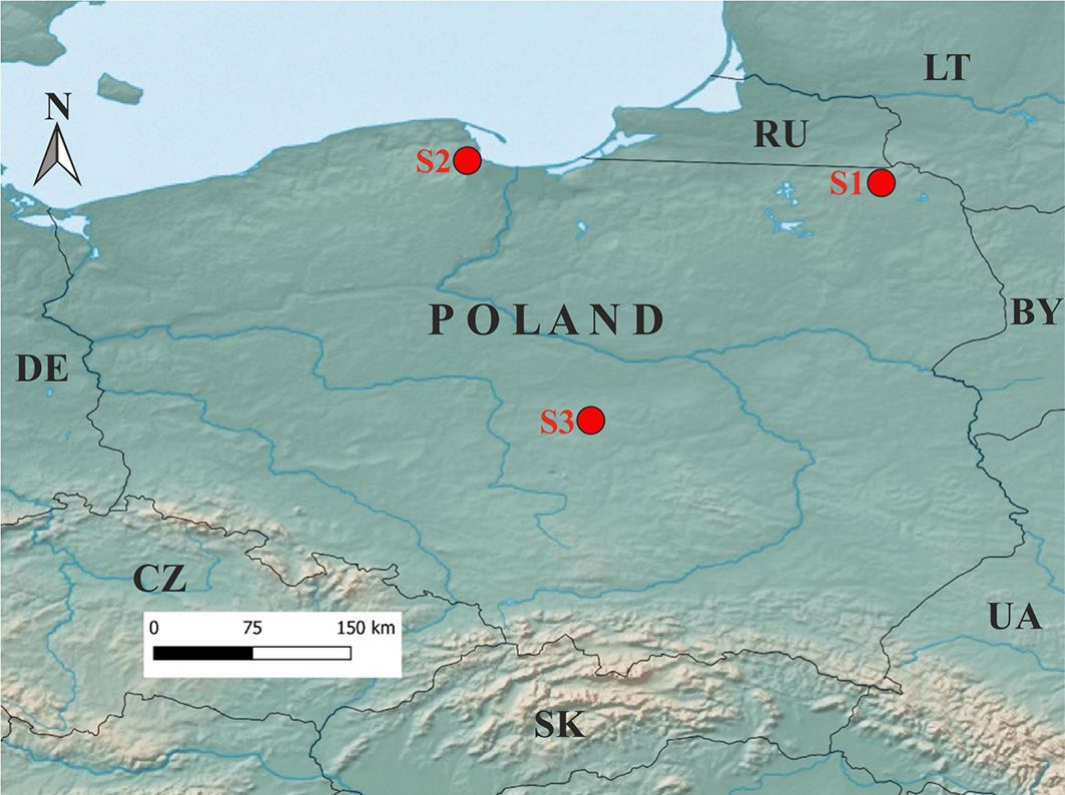
Map showing locations and origins of *Acorus calamus* accessions harvested from different regions of Poland. (S1–S3)—sample code, N—North, BY—Belarus, CZ—Czech Republic, DE—Germany, RU—Russian Federation, SK—Slovakia, UA—Ukraine^55^. The base map of Poland and the neighbouring countries was obtained from http://diva-gis.org/data.

For this study, several samples of *Acorus calamus* rhizomes—both commercially available and collected from natural stands (see Table 4 and Table 5) were analysed. The material from personal collection came from different regions in Poland (see Fig. 8/Table 5) and was authenticated by prof. Justyna Polit from the Faculty of Biology and Environmental Protection of the University of Łódź in Poland. Also, commercially available calamus oil samples were used for the study. The commercially available *A. calamus* rhizomes and oil were obtained from the local herbal shop and came from different producers. The collection of the plant material and related studies complies with relevant institutional, national, and international guidelines and legislation.

**Table 4:** Samples of EOs obtained from natural and commercial resources.

Source	Rhizomes of natural accessions			Commercially available rhizomes			Commercially available EOs		
Code	S1	S2	S3	S4	S5	S6	S7	S8	S9
Country of origin	Poland	Poland	Poland	Hungary	Poland	Poland	Poland	Poland	India

**Table 5:** The origin and harvesting time of the samples obtained from the natural accessions.

Code	Origin (place/region)	Elevation (m amsl)	Latitude (North)	Longitude (East)	Harvesting time
S1	Filipów/Mazury Lake District	180	54°11′36″	22°39′26″	November 2021
S2	Gdańsk/Pomeranian Lake District	140	54°25′11″	18°26′50″	November 2021
S3	Małczew/Greater Poland lowland	230	51°46′40″	19°42′34″	November 2021

First, the personally collected rhizomes were finely cut and dried in the air in the shade. The obtained plant material and the purchased samples of dried rhizomes were subjected to hydrodistillation. Every time fifty grams of plant material were covered with 100 mL of water, sonicated for 15 min at room temperature and subjected to hydrodistillation in Deryng apparatus for 4 h. The obtained essential oil was later collected from the apparatus to a glass vial, dried from water using sodium sulfate, and diluted 100 times with methanol. The extracts were filtered through a nylon syringe filter (pore diameter of 0.22 µm, Sigma Aldrich, St. Louis, MO, USA) and immediately subjected to compositional studies. The purchased essential oil samples were diluted 100 times prior the compositional analysis, filtered and analysed as described above.

### Identification of components in the EO by HPLC-ESI-QTOF-MS/MS

Qualitative analysis of the analysed samples and fractions from CPC analyses was performed by an analytical platform—the HPLC-ESI-QTOF-MS/MS chromatograph 6500 Series (Agilent Technologies, Santa Clara, CA, USA) composed of a binary pump, a degasser, an autosampler, a thermostat, a DAD detector and mass spectrometer (G6530B). The following chromatographic conditions were applied in the study: flow rate of 0.2 mL/min, temperature of 21 °C, injection volume of 2 µL, the UV signals: 280, 254, 210, 230 and 320 nm and the run time of 55 min. The gradient of acetonitrile (solvent B) and water—both with the addition of 0.1% formic acid were used in the following program: 0 min—1% B, 4 min—30% B, 34 min—60% B, 40 min—95% B, 44 min 95% B, 45—1% B. The chromatographic separation was performed on a Zorbax Eclipse Plus RP-18 column (150 mm × 2.1. mm; dp = 3.5 μm) produced by Agilent Technologies (Santa Clara, Ca, USA).

Freshly calibrated and tuned mass spectrometer was operated within the *m/z* range of 40–1200 u, in the further described settings: gas temperature of 250 °C, sheath gas temperature of 300 °C, gas and sheath gas flows of 12 L/min, nebulizer pressure of 35 psig, capillary voltage of 2500 V, nozzle voltage of 1000 V, fragmentor voltage of 100 V, collision energies of 10 and 20 V, skimmer voltage of 65 V. Mass Hunter Workstation Software (v. B.08.00) was used to record and process the collected data. The structure elucidation was based on the comparison of the obtained data with those present in the scientific literature (retention time, high accuracy mass measurement, the analysis of their MS/MS spectra) in a constructed method that aimed at the fragmentation of two the most intensive *m/z* signals in each scan, in two collision energies.

### Quantitative analysis of *β*-asarone content in EO by HPLC-DAD

The quantitative analysis of asarones in EO samples was performed by HPLC-DAD chromatograph produced by Shimadzu (Kyoto, Japan). The instrument was composed of a quaternary pump (LC-20AD), a degasser (DGU-20A), an autosampler (SIL-20A HT), a Photodiode Array Detector (SPD-M20A), and a column oven (CTO-10AS VP). The collection and handling of data was supported by the LabSolutions program. The chromatographic separation was performed on Phenomenex Luna 100 C18 (2) column (150 × 4.6 mm, 5 µm) (Phenomenex, Torrance, CA, USA). The isocratic elution mode of 55% methanol in water was applied (*v/v*). The flow rate was set at 1 mL/min and the detection wavelength used to calculate the content of asarones was selected as 235 nm. First, the concentration of *β*-asarone in the tested samples was calculated based on the calibration curve of the authentic standard. For this purpose a freshly prepared 2 mg/ mL solution of *β*-asarone standard in methanol (95% HPLC purity, Sigma Aldrich, St. Louis, MO, USA) was injected in the concentration range of 0.01–2 mg/mL from 5 individual solutions prepared by the dilution of the stock. The obtained calibration curve equation was calculated as y = 160320853x—5933.5 and the regression coefficient value as R^2^ = 0.9999. Also, the relative amount of asarones (*α-*, *β*- and *γ*-asarone) was calculated based on the HPLC chromatograms.

### Statistical analysis

All the chemometric analyses and visualizations were performed using R v4.2.0^56^ programming language in RStudio^57^ software with pracma^58^, factoextra^59^, and matlib^60^ packages installed.

#### Principal component analysis (PCA)

Before the assessments, all peak areas of the three traced asarones of all nine samples were summed to 100%, hence the amount of every asarone was represented as a percentage share of a total detector response of a given source (Table 2). After the percentage rescaling of samples, all the variables (i.e. *α*-, *β*- and *γ*-asarone) were autoscaled in order to enable a formal decomposition of the covariance matrix. As a result of the application of standard principal component analysis (PCA) algorithm, first two principal components (PCs) were considered relevant in the presented case. In the next step, their vectors were rotated in space in order to maximize values of correlation coefficients between the original variables and the two orthogonal factors using VARIMAX approach. The linear map of samples (sources) in the space of the resulting varivectors (referred to as ‘dimensions’ in the main text) was prepared by multiplying the matrix of original variables’ loadings in the space of varivectors by the matrix of the autoscaled dataset (see Tables S2 and S3 in the Supplementary File).

#### Hierarchical clustering

Hierarchical clustering for the studied asarones’ case was executed using Ward method, basing on a standard matrix of Euclidean distances between the sources.

### Centrifugal partition chromatography method of removing *β*-asarone

#### Biphasic solvent system selection

The biphasic solvent system to be used for the fractionation of *Acorus calamus* EO by means of Centrifugal Partition Chromatography was established by determining its partition coefficient (Kd) in several biphasic solvents mixtures, using the shake-flask method. The selection criteria for the system were based on the differences in partition coefficient values of single constituents of the extract and the selectivity (**α**) of every system for *α*-asarone and *β*-asarone. These parameters were determined as follows: 4.95 mL of upper and lower phases of the corresponding solvent system were placed in a test tube and 0.1 mL of the *Acorus calamus* EO was added. The tubes were vigorously shaken and after equilibrium was achieved, both phases were separately filtered through a syringe filter (pore diameter of 0.22 µm) into separate vials and analysed by HPLC-DAD using the chromatographic conditions described in section *Quantitative analysis of β-asarone content.*

The Kd values of *α*-asarone and *β*-asarone were defined as the peak area of the corresponding compound in the lower phase divided by the peak area of the compound in the upper phase. Next, Kd ratios of each compound were calculated to determine the selectivity (**α)** of each solvent system, according to Ito and Golden^44^ and the formula below.

1 $$\alpha = Kd1/Kd2$$

where: *Kd1*, *Kd2*—partition coefficient of two asarones, where *Kd1* > *Kd2*.

#### Acorus calamus EO fractionation

The removal of *β*-asarone from the essential oil was performed by means of centrifugal partition chromatography (CPC) on a Gilson CPC-250 apparatus (Gilson Inc., Middleton, WI, USA), equipped with the Ecom flash 14 DAD 600 detector (Gilson Inc., Middleton, WI, USA) and Gilson fraction collector LS-5600 (Gilson Inc., Middleton, WI, USA). The biphasic liquid system HEMWat/ Arizona X (n-hexane/EtOAc/MeOH/water, 9: 1: 9: 1 *v*/*v*/*v/v*) was prepared by mixing the respective portions of the solvents at room temperature. The separation was performed in ascending mode, using the upper phase as the mobile phase. First, the lower phase was loaded to the column at a flow rate of 30 mL/min, at rotational speed of 500 rpm and the time of 12 min. Next the upper phase was pumped through the stationary phase for the following 13 min at a flow rate of 5 mL/min, rotational speed of 2200 rpm, until equilibrium was achieved with a pressure of 50 bar. The stationary phase retention (*Sf*) was determined according to Berthod and Faure^45^ and the equation below.

2 $$Sf = Vs/Vc$$

where: *Vs*—retained volume of stationary phase in the column after the equilibrium; *Vc*—column volume.

Then, 1 mL of the *Acorus calamus* essential oil, dissolved in 9 mL of equal volumes of upper and lower phase (1:1, *v/v*), was introduced via manual injection valve and an injection loop of 10 mL. The upper phase was pumped at the analytical conditions during 150 min, while fractionation occurred (12 mL/fraction). Afterwards, the lower phase was introduced to the column at a flow rate of 30 mL/min and a rotational speed of 500 rpm during 10 min, as an extrusion step, totalizing 160 min of the fractionation. The effluent was monitored at a wavelength of 254 nm into 12 mL volume vessels. Data were collected and processed with Gilson Glider CPC V5.1d.01 software. The composition of fractions was confirmed by HPLC-ESI-QTOF-MS/MS instrumentation, whereas their purity with help of HPLC-DAD apparatus according to the previously described protocol. All the fractions except those corresponding to asarones were combined and the solvents evaporated under reduced pressure at the temperature not exceeding 40 °C.

## Supplementary Information

Supplementary Information.

## Data Availability

Most of the data generated or analyzed during this study are included in this published article (and its Supplementary Data files).
