# Supplementary material for: The role of centrifugal partition chromatography in the removal of β-asarone from Acorus calamus essential oil
Source: Sci Rep. 2022 Dec 23;12:22217. doi: 10.1038/s41598-022-26726-6 (PMC9789137; doi:10.1038/s41598-022-26726-6)
Supplement: Supplementary file 1 — Supplementary Information. [file 41598_2022_26726_MOESM1_ESM.docx]

**SUPPLEMENTARY FILE**

Table S1. The MS/MS fragmentation spectra of the components that were identified in the studied *Acorus calamus* essential oil by HPLC-MS

| **2-Allyl-5-ethoxy-4-methoxyphenol isomer 1** |  |
| --- | --- |
| **2-Allyl-5-ethoxy-4-methoxyphenol isomer 2** |  |
| **2-Allyl-5-ethoxy-4-methoxyphenol isomer 3** |  |
| **a-Cedrene** |  |
| **Acoronene** |  |
| **Acorusnol** |  |
| Asaronaldehyde |  |
| **Aspidinol** |  |
| Calamenene |  |
| Calamenene isomer |  |
| **Calarene** |  |
| **Gluconic acid** |  |
| **Isoacoramone** |  |
| Methyl eugenol |  |
| **Shyobunone** |  |
| **Squamulosone isomer 1** |  |
| **Squamulosone isomer 2** |  |
| **Isoacoramone isomer2** |  |

**Table S2.** Original variables’ loadings in the space of the first two dimensions (varivectors, i.e. principal components after the VARIMAX rotation).

|  | Dimension 1 | Dimension 2 |
| --- | --- | --- |
| β-asarone | -0,99991 | 0,013181 |
| γ-asarone | -0,02275 | 0,999741 |
| α-asarone | 0,995484 | -0,09493 |

**Table S3.** Samples’ (=sources’) scores in the space of the first two dimensions.

|  | Dimension 1 | Dimension 2 |
| --- | --- | --- |
| S4 | 1,226921 | 0,404046 |
| S5 | 0,354334 | 0,13808 |
| S6 | 3,900486 | 0,564937 |
| S7 | -2,46135 | 0,855178 |
| S8 | -1,47784 | 1,038685 |
| S9 | -2,41469 | 0,677813 |
| S1 | 0,96468 | -0,70188 |
| S2 | -0,10416 | -0,94954 |
| S3 | 0,011629 | -2,02732 |

**Table S4**. Purity of the isolates after CPC fractionation.

| *Sample* | | Standard EO | *α* – asarone fraction | *β* – asarone fraction |
| --- | --- | --- | --- | --- |
| *Asarone content [%]* | α | 4.7 | **93.7** | 3.5 |
|  | β | 94.1 | 1.0 | **95.5** |
|  | γ | 1.1 | 2.2 | 1.0 |
